# Intraductal oncocytic papillary neoplasm of the pancreas: clinical and radiological features compared to those of intraductal papillary mucinous neoplasm

**DOI:** 10.1007/s00261-023-03985-z

**Published:** 2023-06-26

**Authors:** Moto Nakaya, Yudai Nakai, Mai Takahashi, Yoshihiko Fukukura, Keisuke Sato, Arisa Kameda, Yuki Tashiro, Sakiko Kageyama, Keitaro Sofue, Tsubasa Nakano, Kengo Yoshimitsu, Nagaaki Marugami, Nobuyuki Takeyama, Mariko Tanaka, Kiyoshi Hasegawa, Takeyuki Watadani

**Affiliations:** 1 Department of Radiology, Graduate School of Medicine, The University of Tokyo, 7-3-1 Hongo, Bunkyo-ku, Tokyo 113-8655 Japan; 2 Department of Radiology, Kobe University Graduate School of Medicine, 7-5-2 Kusunoki-cho, Chuo-ku, Kobe City, Hyogo 650-0017 Japan; 3 Department of Radiology, Kagoshima University Graduate School of Medical and Dental Sciences, 8-35-1 Sakuragaoka, Kagoshima City, Kagoshima 890-8544 Japan; 4 Department of Radiology, Faculty of Medicine, Fukuoka University, 7-45-1 Nanakuma, Jonan-ku, Fukuoka City, Fukuoka 814-0180 Japan; 5 Department of Radiology, Nara Medical University, 840 Shijo-cho, Kashihara, Nara City, Nara 634-8522 Japan; 6 Department of Radiology, Showa University Fujigaoka Hospital, 1-30 Fujigaoka Aoba-ku, Yokohama-shi, Kanagawa 227-8501 Japan; 7 Department of Radiology, Tohoku University School of Medicine, Tohoku University Hospital, 1-1 Seiryocho, Aoba-ku, Sendai City, Miyagi 980-8574 Japan; 8 Department of Pathology, Graduate School of Medicine, The University of Tokyo, 7-3-1 Hongo, Bunkyo-ku, Tokyo 113-8655 Japan; 9 Hepato-Biliary-Pancreatic Surgery Division, Department of Surgery, Graduate School of Medicine, The University of Tokyo, 7-3-1 Hongo, Bunkyo-ku, Tokyo 113-8655 Japan

**Keywords:** Computed tomography, Magnetic resonance imaging, Fluorine-18-2-deoxy-D-glucose positron emission tomography, Intraductal oncocytic papillary neoplasm, Intraductal papillary mucinous adenoma, Intraductal papillary mucinous carcinoma

## Abstract

**Purpose:**

This study aimed to characterize the clinical and imaging findings of intraductal oncocytic papillary neoplasm of the pancreas (IOPN-P) compared to those of intraductal papillary mucinous adenoma/carcinoma (IPMA/IPMC).

**Methods:**

This multi-institutional retrospective study reviewed the clinical, imaging, and pathological findings of 21 patients with pathologically proven IOPN-P. Twenty-one computed tomography (CT) and magnetic resonance imaging, and seven ^18^F-fluorodeoxyglucose (FDG)-positron emission tomography were performed before surgery. The following findings were evaluated: preoperative blood test results, lesion size and location, pancreatic duct diameter, contrast-enhancement effect, bile duct and peripancreatic invasion, maximum standardized uptake (SUVmax) value, and pathological stromal invasion.

**Results:**

Serum carcinoembryonic antigen (CEA) and cancer antigen 19-9 (CA19-9) levels were significantly higher in the IPMN/IPMC group than in the IOPN-P group. Except in one patient, IOPN-P showed multifocal cystic lesions with solid components or a tumor in the main pancreatic duct (MPD) with dilatation. IOPN-P had a higher frequency of solid parts and a lower frequency of downstream MPD dilatation than IPMA. IPMC showed smaller overall cyst size, more radiological peripancreatic invasion, and worse recurrence-free and overall survival than IOPN-P. The average SUVmax value of IOPN-P was 7.5. Pathologically, 17 of the 21 IOPN-Ps had a malignant component, and six showed stromal invasion.

**Conclusion:**

IOPN-P shows cystic-solid lesions similar to IPMC but has lower serum CEA and CA19-9 levels, larger overall cyst size, lower frequency of peripancreatic invasion, and more favorable prognosis than IPMC. Moreover, the high FDG uptake by IOPN-Ps may be a characteristic finding of this study.

**Graphical abstract:**

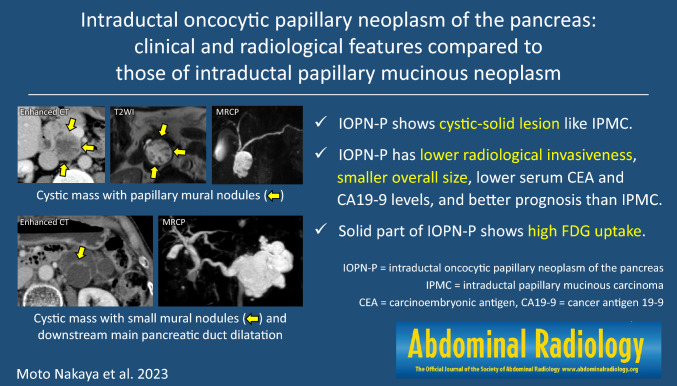

## Introduction

Intraductal oncocytic papillary neoplasm of the pancreas (IOPN-P) is a pancreatic neoplasm characterized by papillary structures lined with eosinophilic cells and was first described by Adsay et al. [1]. Although intraductal papillary mucinous neoplasm (IPMN) was histologically divided into four subclasses in the fourth edition of the World Health Organization (WHO) Classification in 2010, IOPN-P became independent in the revised fifth edition of the WHO Classification in 2019 [2]. IOPN-P has fewer mutations in *KRAS*, *GNAS*, and *RNF43* than the other three subtypes (gastric, intestinal, and pancreatobiliary) of IPMN [3]. Furthermore, pancreatic and bile duct IOPNs harbor recurrent *ATP1B1-PRKACB*, *DNAJB1-PRKACA*, or *ATP1B1-PRKACA* fusion genes, which do not occur in other tumors of the pancreatobiliary system [4]. Therefore, although IOPN-P and IPMN are macroscopically similar, they are pathologically and genetically recognized as different [2]. For instance, IOPN-P generates lower quantities of mucin and commonly assembles into intricate conglomerations comprising both cystic and solid elements, frequently resulting in the radiological identification of pancreatic ductal adenocarcinoma [5]. Although IOPN-P has unique pathological characteristics, little information is available regarding its imaging characteristics [6, 7]. This study aimed to characterize computed tomography (CT), magnetic resonance imaging (MRI), and clinical findings of IOPN-P compared to those of IPMN.

## Methods

### Patient profiles

At the 35th Annual Meeting of the Japanese Society of Abdominal Radiology held in June 2022, patients with pathologically proven IOPN-P from seven institutions were presented and included in this study. This retrospective multicenter study was approved by all the institutional review boards, and the requirement for written informed consent was waived. The mean age of the 21 (15 man, six women) patients at diagnosis was 66.3 (range, 36–85) years. All the patients underwent surgery. Preoperative CT, MRI, and ^18^F-fluorodeoxyglucose-positron emission tomography (FDG-PET) were performed in 21, 21, and seven patients, respectively. In addition, from the pathological database of the University of Tokyo Hospital, 20 consecutive intraductal papillary mucinous adenomas (IPMAs) and 20 consecutive intraductal papillary mucinous carcinomas (IPMCs) from January 2013 to December 2016 were selected for comparison. Patients with IPMA/IPMC who did not undergo contrast-enhanced CT or MRI were excluded. All patients were anonymized.

### Clinical data

The medical records of the patients were reviewed for clinical findings. We extracted data on patient demographics, preoperative laboratory data (serum white blood cell count, C-reactive protein, and amylase levels), and tumor markers such as carcinoembryonic antigen (CEA) and carbohydrate antigen 19-9 (CA19-9). Tumor recurrence was determined based on the imaging findings.

### Image acquisition

Abdominal CT was performed using a multi-detector CT unit (LightSpeed QX/i or Discovery CT750 HD; GE Healthcare, Milwaukee, WI, USA; SOMATOM Definition: Siemens, Erlangen, Germany; Aquilion ONE, Aquilion PRIME, Aquilion 64, Aquilion 16, Aquilion 320, or Aquilion Precision: Canon/Toshiba, Otawara, Japan; or IQon Spectral CT: Philips Healthcare, Best, the Netherlands) with a tube voltage of 120 kVp. Contrast-enhanced CT was not performed in one patient with IOPN-P because of a history of adverse reactions to iodine contrast media. After intravenous contrast medium administration, contrast-enhanced CT acquisition timing included the arterial (approximately 40 s) and portal (60–90 s) phases. Arterial phase images were not obtained in three patients. An automated power injector was used to inject 100–135 mL or 600–650 mg of iodine/kg body weight at a rate of 2.0–4.0 mL/s. CT images with a thickness 5 mm or less were used for evaluation.

MRI examinations were performed using 1.5-T (SIGNA HDxt: GE, Milwaukee, WI, USA; MAGNETOM Avanto, MAGNETOM VISION: Siemens, Erlangen, Germany; or Ingenia, Achieva: Philips Medical Systems, Best, the Netherlands) or 3.0-T (MAGNETOM Skyra, MAGNETOM Trio A Tim, or MAGNETOM Prisma: Siemens, Erlangen, Germany; Achieva D-Stream: Philips Medical Systems, Best, the Netherlands; Titan: Canon, Otawara, Japan; or Discovery MR750w or Signa HDXt: GE, Milwaukee, WI, USA) MRI scanners. Axial dual-echo T1-weighted images (repetition time [TR], 4.1–256 ms; echo time [TE], 1.1–5.3 ms; slice thickness, 3.5–8.0 mm), axial and coronal T2-weighted images (TR, 543–8000 ms; TE, 54–320 ms; slice thickness, 4.0–6.0 mm), and magnetic resonance cholangiopancreatography images (TR, 800–6000 ms; TE, 105–900 ms; slice thickness, 1.4–5.0 mm) were obtained.

FDG-PET/CT or FDG-PET/MRI was performed in seven patients (Discovery 690, Discovery MI, Discovery MI DR, or SIGNA PET/MR: GE, Milwaukee, WI, USA; or Aquiduo:Toshiba/Siemens Co., Ltd.) 1 h after the administration of 2.96–4.38 MBq/kg FDG at with noncontrast CT of 2.0–3.75 mm thickness.

### Image analysis

All images were evaluated by a radiologist with 5 years of experience in abdominal radiology under the supervision of a board-certified radiologist with 10 years of experience.

The following features were evaluated using CT and/or MRI: the localization of the lesion; main pancreatic duct (MPD), branch duct or mixed type; diameter of the entire cyst, including the intracystic solid part (measurement of the MPD type was not performed); diameter of the solid portion; contrast enhancement effect (CT values); bile duct obstruction; maximum diameter of the MPD; and dilatation of the MPD downstream of the tumor. This was a multi-center study in which the time of the arterial phase was different; hence, we did not evaluate the CT values of solid portion in the arterial phase. We also evaluated lymph node enlargement, peripancreatic soft tissue invasion, and dilatation of the MPD due to obstruction. Peripancreatic invasion was evaluated with reference to a previous study [8]. The maximum standardized uptake (SUVmax) value of the solid part of the tumor was measured.

### Pathological analysis

Initial pathological diagnoses were established by board-certified pathologists at each institution. Information on malignant changes and stromal invasion was extracted from the pathological records.

### Statistical analyses

Statistical analyses were performed using EZR software (EZR version 1.55; Jichi Medical University Saitama Medical Center, Saitama, Japan) [9]. Fisher’s exact test was used to evaluate the association between two and three categorical variables. To adjust for multiple comparisons, the Holm method was used to control for the family wise error rate. In addition, we performed a one-way analysis of variance or the Kruskal–Wallis test to compare the means or medians of the three groups, depending on whether they were normally distributed. The Shapiro–Wilk test was used to assess whether a continuous variable followed a normal distribution. To determine the group(s) differed significantly from each other, we performed a post-hoc analysis using Tukey’s test (after one-way analysis of variance) or the Steel–Dwass test (after the Kruskal–Wallis test). Overall survival and recurrence-free survival were calculated from the pancreatic resection date and compared using the log-rank test. All *P* values were two-sided, and *P* <.05 was considered significant.

## Results

### Clinical findings

The demographic and clinical characteristics of the study population are summarized in Table 1. The median follow-up periods were 1301 (range, 45–5112), 2941 (range, 608–4210), and 1187 (range, 420–4621) days in patients with IOPN-P, IPMA, and IPMC, respectively. In patients with IOPN-P, information on recurrence and death was not available for five patients, and three (3/16, 19%) recurrences and one (1/16, 6%) death occurred. No recurrence or death occurred in the patients with IPMA. Among the patients with IPMC, 13 (13/20, 65%) had recurrences and 12 (12/20, 60%) died. Patients with IOPN-P had better recurrence-free survival (*P*=0.01, log-rank test) and overall survival (*P*=0.02, log-rank test) than those with IPMC. One patient with IOPN-P was excluded from the statistical analysis because the timing of recurrence was unknown.

**Table 1:** Demographic, clinical, radiological, and pathological characteristics of patients

	No. of patients with IOPN-P evaluated	IOPN-P	IPMA	*P* value	IPMC	*P* value
Mean age (years)^*^	21	66.3 (36–85)	70.3 (60–80)	0.32	72.5 (53–85)	0.07
Sex (M:F)^†^	21	15:6	12:8	1.0	10:10	0.62
Mean serum WBC level (/μL)^*^	18	5627 (2800–9600)	5855 (2900–8600)	0.91	5690 (2800–9500)	0.99
Mean serum CRP level (mg/dL)^‡^	18	0.41 (0.01–3.14)	0.28 (0.01–2.61)	0.98	0.31 (0.01–3.71)	0.99
Mean serum amylase level (U/L)^‡^	18	104 (20–412)	124 (44–431)	0.19	82.0 (17–176)	0.93
Mean serum CEA level (ng/mL)^‡^	16	0.45 (0.01–3.14)	4.81 (1.3–13.8)	**<0.001**	4.82 (2.2–9.8)	**< 0.001**
Serum CEA > 5 ng/mL^†^	16	0/16	5/20	0.11	6/20	0.07
Mean serum CA19-9 level (U/mL)^‡^	17	6.6 (0.7–57.4)	23.8 (1–156)	**0.04**	82.0 (1–483)	**< 0.001**
Serum CA19-9 > 37 U/mL^†^	17	1/17	3/20	0.61	7/20	0.14
Localization of the lesion (pancreatic head:body:tail)^†^	21	10:2:9	11:5:4	0.48	10:2:8	1.00
Type (main pancreatic duct:branch duct:mixed)^†^	21	1:13:7	2:6:12	0.57	0:8:12	0.48
Mean diameter of entire cyst, including intracystic solid part (mm)^‡^	18	51.7 (16–140)	34.7 (20–53)	0.08	27.0 (5–70)	**0.04**
Existence of the solid portion^†^	21	20/21	5/20	**<0.001**	19/20	1.00
Mean diameter of the solid portion (mm)^‡^	18	24.1 (2.8–87)	3.85 (10–34)	**<0.001**	20.6 (7–42)	0.89
Mean plain CT values of the solid portion (HU)^‡^	16	31.8 (14–57)	31.0 (25–44)	1.0	36.1 (23–49)	0.26
Mean CT values of the solid portion in the portal phase (HU)^‡^	16	79.3 (38–127)	73.5 (65–93)	0.61	85.5 (54–123)	0.77
Bile duct obstruction^†^	21	2/21	0/20	1.0	2/20	1.00
Mean maximum diameter of the main pancreatic duct (mm)^‡^	19	7.39 (2–18)	9.1 (3–19)	0.37	8.05 (3–24)	0.73
Dilatation of main pancreatic duct downstream^†^	21	6/21	18/20	**<0.001**	10/20	0.21
Dilatation of the main pancreatic duct due to tumoral obstruction^†^	19	4/19	1/20	0.35	9/20	0.35
Peripancreatic soft tissue invasion^†^	19	3/19	0/20	0.11	12/20	**0.02**
Lymph node enlargement^†^	21	0/19	0/20	1.0	2/20	0.70
Pathological stromal invasion^†^	19	6/19	0/20	**0.008**	18/20	**< 0.001**

Bold values indicate statistical significance

*CEA* carcinoembryonic antigen, *CRP* C-reactive protein, *F* female, *HU* Hounsfield unit, *IOPN-P* intraductal oncocytic papillary neoplasm of the pancreas, *IPMA* intraductal papillary mucinous adenoma, *IPMC* intraductal papillary mucinous carcinoma, *M* male, *WBC* white blood cell

*One-way analysis of variance with post-hoc Tukey test

^†^Fisher’s exact test using the Holm method

^‡^Kruskal–Wallis test with the post-hoc Steel–Dwass test

### Radiological findings

**Fig. 1:**
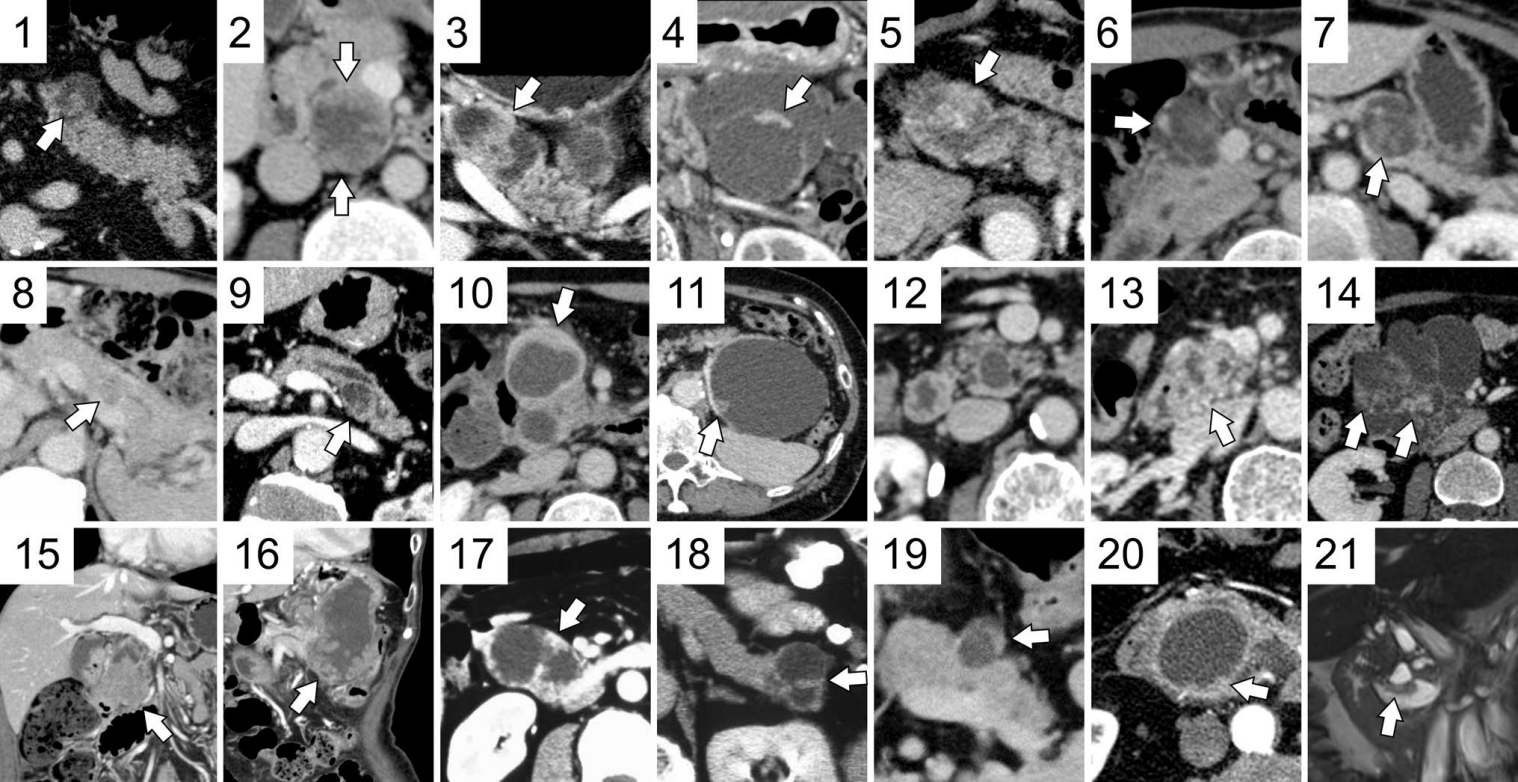
Cases 1–21 of intraductal oncocytic papillary neoplasm of the pancreas are listed with contrast-enhanced computed tomography images. Arrows show solid parts. Coronal views show the location of duodenal involvement in Case 15 and gastric and transverse colon involvement in Case 16. In Case 21, a coronal image of steady-state free precession has been presented because contrast-enhanced computed tomography was not obtained.

**Fig. 2:**
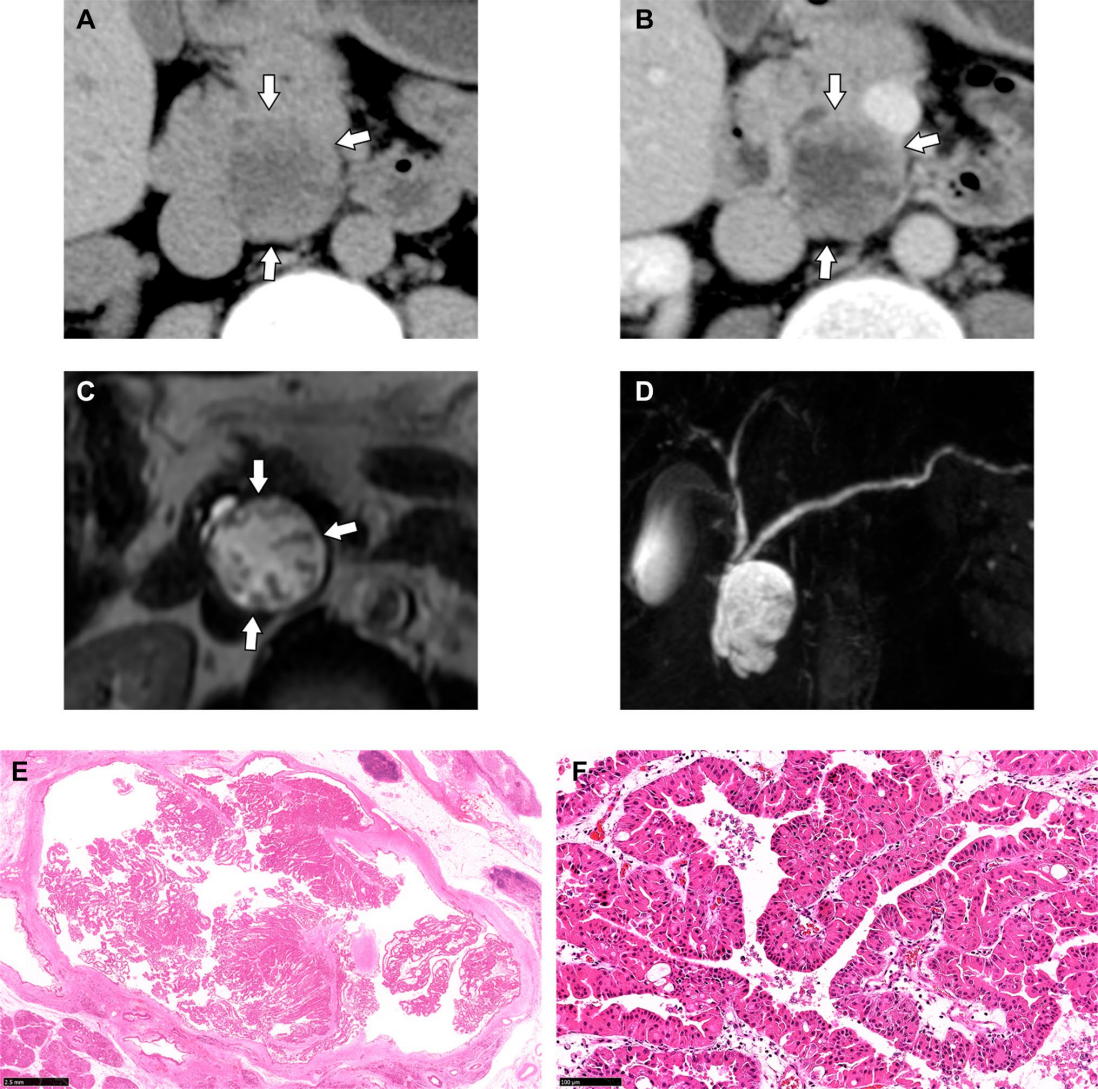
Case of a 36-year-old man with a cystic mass in the pancreatic head found on abdominal ultrasonography during a health checkup (Case 2). The well-demarcated cystic mass with multiple papillary mural nodules was observed in the head of the pancreas (**a**, plain computed tomography;**b**, contrast-enhanced computed tomography;**c**, T2-weighted image: arrow). The solid portion shows a contrast enhancement equivalent to that of the pancreas parenchyma in the parenchymal phase of contrast-enhanced computed tomography (**b**). On T2-weighted image, papillary nodules show slightly higher signal intensity than the pancreatic parenchyma (**c**). Magnetic resonance cholangiopancreatography confirms the mass as a branch duct type (**d**). Microscopically, the tumors form arborizing papillae with delicate to edematous fibrovascular cores within a branch of the pancreatic duct (**e**: hematoxylin-eosin stain, ×8.5). Tumor cells show oncocytic features with abundant eosinophilic granular cytoplasm, interspersed with a small number of goblet cells (**f**: hematoxylin-eosin stain, ×200). No stromal invasion was observed.

**Fig. 3:**
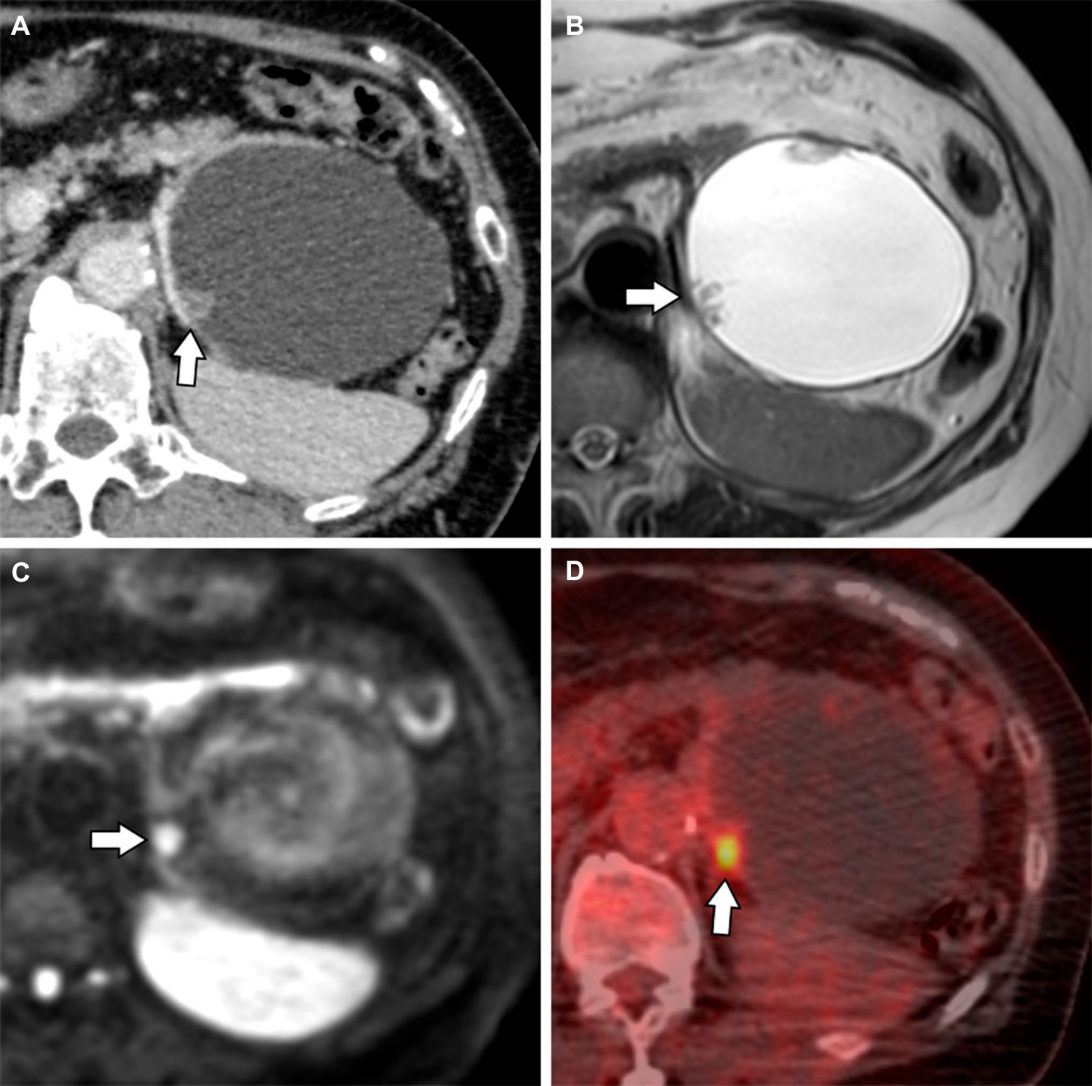
Case of a 66-year-old woman with a chief complaint of abdominal distension shows a mass in the tail of the pancreas (Case 11). A 91-mm-diameter cystic lesion is observed in the tail of the pancreas, and a contrast-enhanced 10-mm-diameter mural nodule is observed on contrast-enhanced computed tomography (**a**: arrow). On T2-weighted and diffusion-weighted images, mural nodule shows high signal intensity (**b**,**c**: arrow). ^18^F-fluorodeoxyglucose (FDG)-positron emission tomography/computed tomography shows accumulation in the mural nodule (**d**: arrow).

**Fig. 4:**
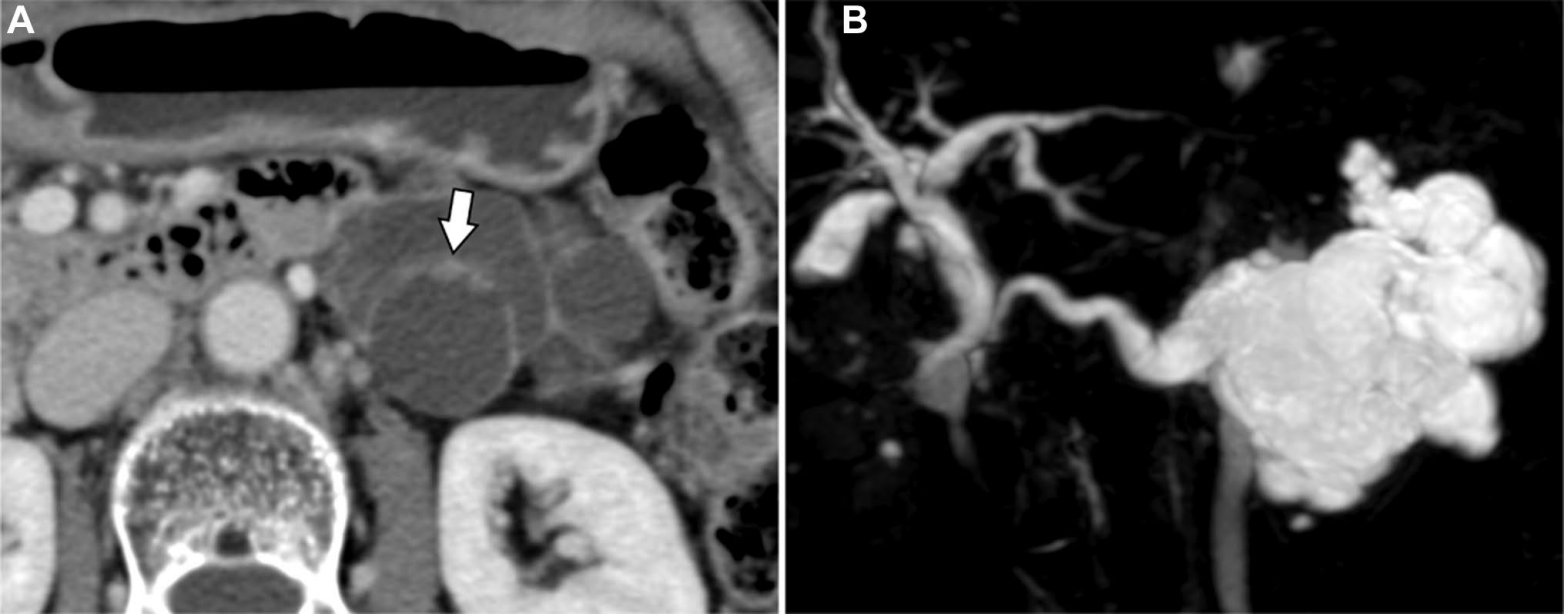
Case of an 80-year-old woman with a cystic mass in the pancreatic tail found on abdominal ultrasonography during a health checkup (Case 4). A well-demarcated multilocular cystic lesion with a small mural nodule is found in the tail of the pancreas on contrast-enhanced computed tomography (**a**: arrow). The solid portion shows a stronger contrast enhancement than the pancreas in the portal phase of contrast-enhanced computed tomography. Magnetic resonance cholangiopancreatography confirms the mass as a mixed type with prominent pancreatic duct dilatation downstream of the mass (**b**).

Fig. 1 shows the images of all patients, and Fig. 2, 3, 4, 5 show the details of the representative cases. Figs. 6 and 7 shows representative cases with IPMA and IPMC, respectively. Because digital data for all sections were not available in two patients, their images could only be evaluated qualitatively. Case 16 has been submitted to another journal as a case report.

**Fig. 5:**
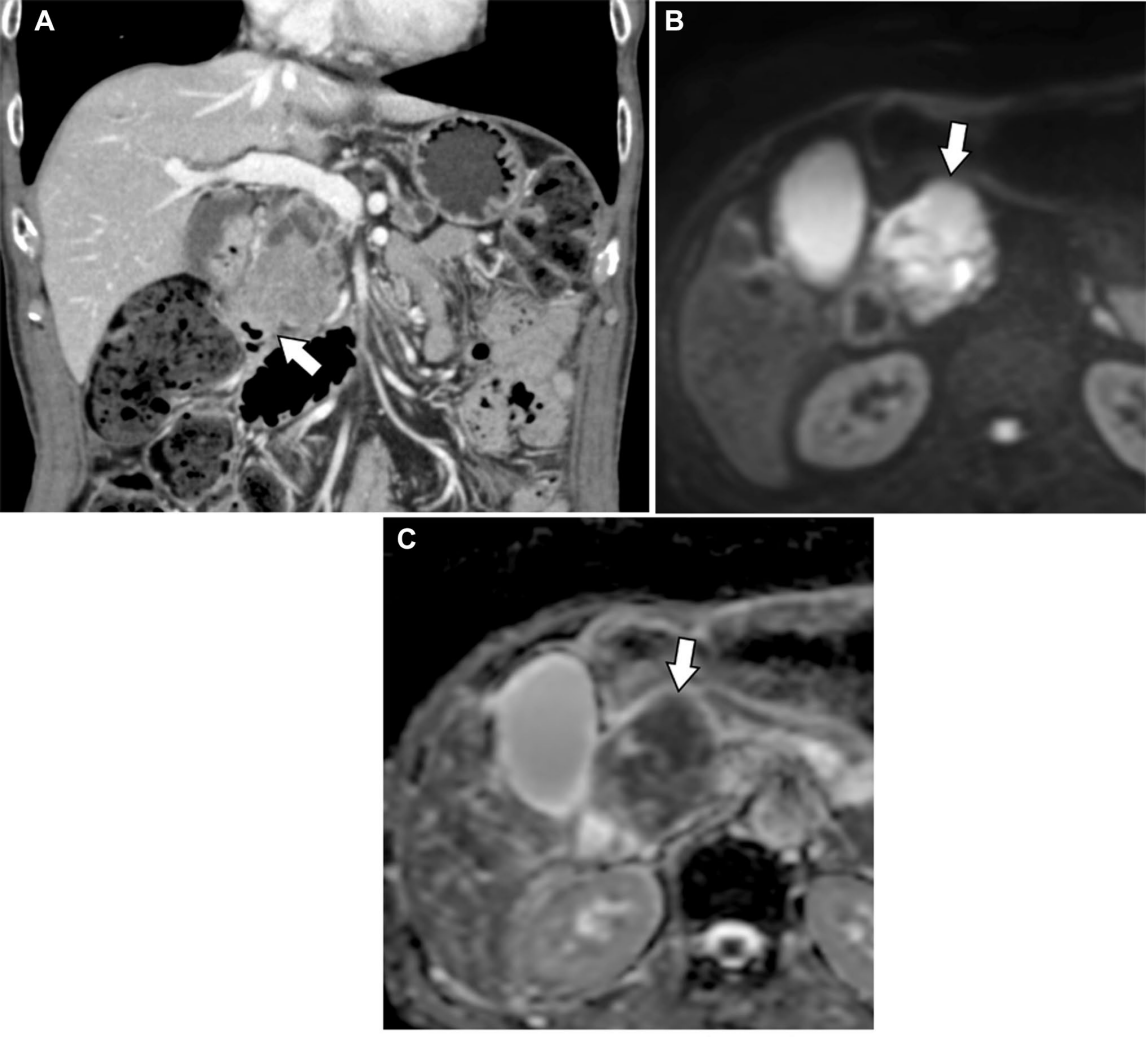
Case of a 72-year-old man with a chief complaint of weight loss shows a mass in the head of the pancreas (Case 15). Coronal contrast-enhanced computed tomography reveals a pancreatic head mass invading the duodenum (**a**: arrow). On diffusion-weighted image, the tumor shows high intensity (**b**: arrow), and the apparent diffusion coefficient value is low (1.02 × 10^–3^ mm^2^/s) (**c**: arrow). Pathological examination reveals the tumor has invaded the proper muscular layer of the duodenum and accessory papilla.

**Fig. 6:**
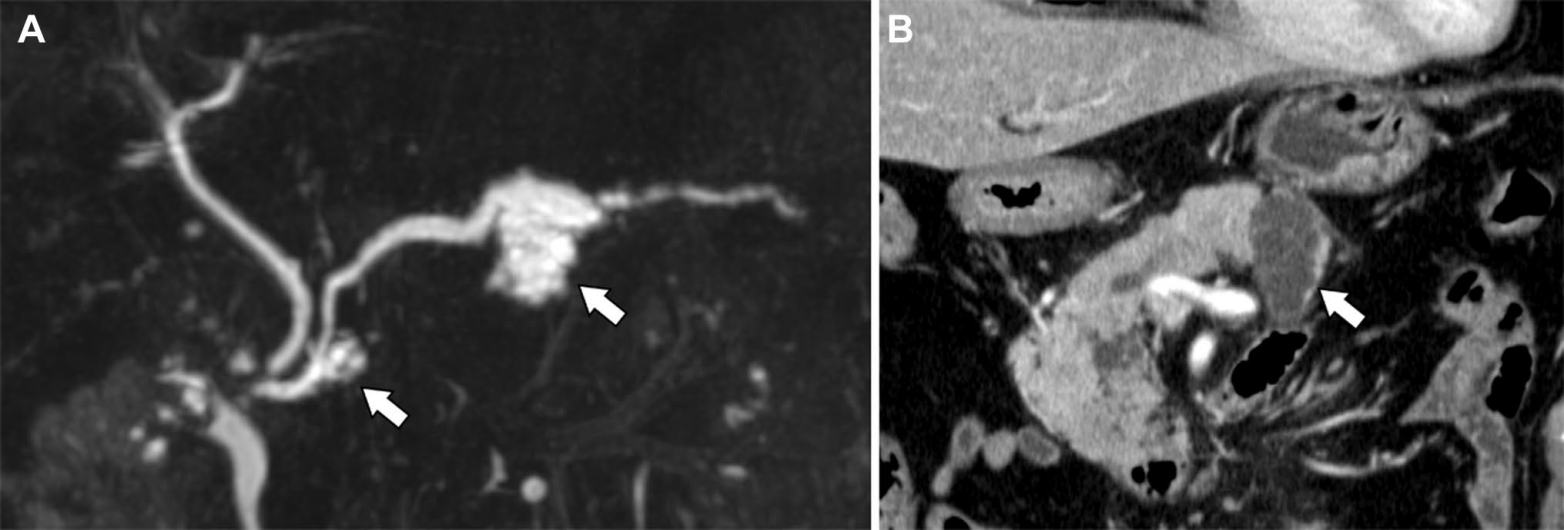
Case of a 74-year-old man suffering from recurrent pancreatitis. Magnetic resonance cholangiopancreatography shows multilocular cystic mass in the head and body of the pancreas (**a**: arrow). Main pancreatic duct is dilated without obstruction. On coronal contrast-enhanced computed tomography, cystic lesion in the body of the pancreas has no solid component (**b**: arrow). Both were resected and pathologically diagnosed as intraductal papillary mucinous adenoma.

**Fig. 7:**
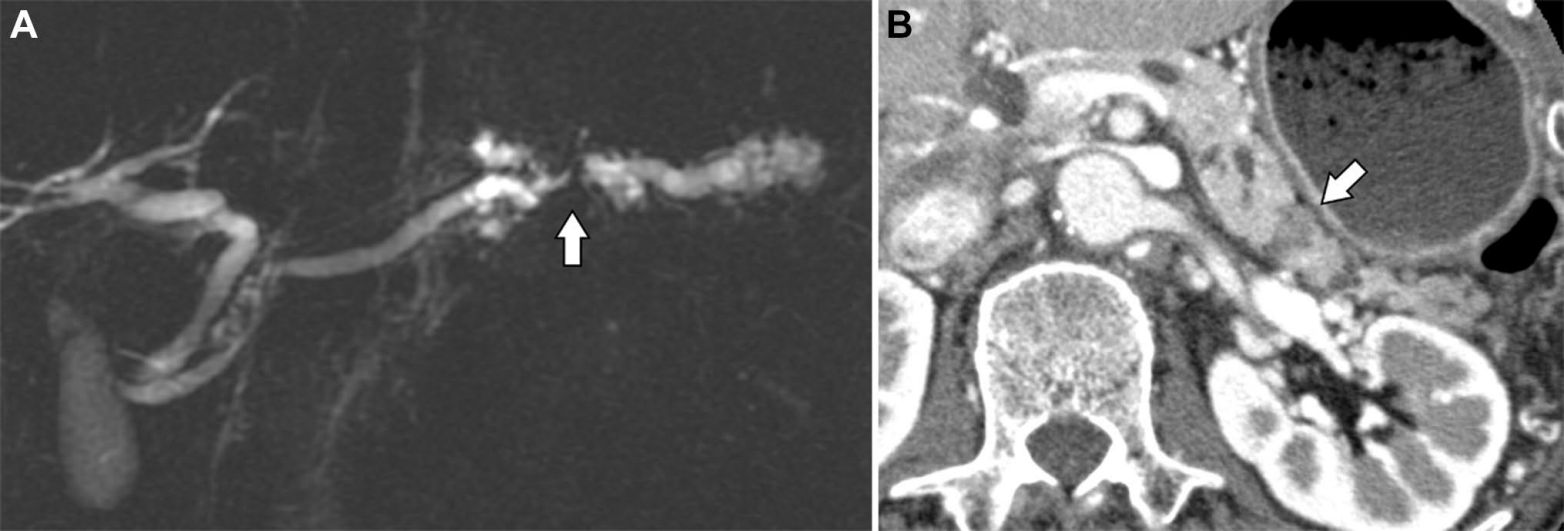
Case of a 70-year-old man under follow-up for intraductal papillary mucinous neoplasm. Magnetic resonance cholangiopancreatography shows obstruction of the main pancreatic duct at the pancreatic tail (**a**: arrow). Dilatation of the main pancreatic duct downstream of the obstruction suggests mucus production. In the late arterial phase on computed tomography, a hypovascular tumor shows peripancreatic invasion (**b**: arrow). It was resected and pathologically diagnosed as intraductal papillary mucinous carcinoma with peripancreatic invasion.

The tumors were located in the pancreatic head (*n* = 10), body (*n* = 2), or tail (*n* = 9), with the MPD type (*n* = 1), branch duct type (*n* = 13), or mixed type (*n* = 7). The mean diameter of the entire cyst, including the intracystic solid part, was 50.0 (range, 16–140) mm; the mean diameter of the solid portion was 24.1 (range, 2.8–87) mm; and the mean maximum diameter of the MPD was 7.4 (range, 2–18) mm. The mean CT values were 31.8 (range, 14–57) Hounsfield unit (HU) for the solid portion and 79.3 (range, 38–127) HU in the portal phase. Bile duct obstruction (2/21), dilatation of the downstream MPD (6/21), and enlarged lymph nodes (0/19) were also observed. On FDG-PET, the tumor revealed FDG uptake in the solid component in all six patients, and FDG uptake was not observed in one patient without a solid component. The mean SUVmax value of the six patients with IOPN-Ps on average was 7.5 (range, 3.74–10.2). Comparisons between IPMA and IPMC are summarized in Table 1.

### Pathological findings

All IOPN-Ps showed intraductal growth with a complex architecture with arborizing papillae. The papillae were lined by cuboidal or columnar cells with oncocytic eosinophilic granular cytoplasm. Pathological data were lost and could not be reevaluated in two patients. Seventeen (17/19) patients had malignancy, and six (6/19) had stromal invasion. Eighteen (18/20) patients with IPMC had stromal invasion.

## Discussion

To the best of our knowledge, this is the first study to compare the imaging and clinical findings between IOPN-P and IPMA/IPMC. Except in one patient, IOPN-P showed multifocal cystic lesions with solid components or a tumor in the MPD with dilatation, which resembled IPMC. IOPN-P had a higher frequency of solid parts (*P* < 0.001) and a lower frequency of downstream MPD dilatation (*P* < 0.001) than IPMA. IPMC showed smaller overall cyst size (*P* = 0.04), higher frequency of radiological peripancreatic invasion (*P* = 0.02), and worse recurrence-free and overall survival than IOPN-P. Serum CEA and CA 19-9 levels were higher in IPMN/IPMC than in IOPN-P.

The mean age of patients with IOPN-P in previous literature with imaging reports was approximately 60 years [5, 10, 11], and the reported tumor size for the entire disease varied from 20 to 180 mm [6]. The mean age and size of IOPN-Ps in the 21 patients in this study were similar to those reported previously.

In the present study, we evaluated the CT, MRI, and FDG-PET findings of IOPN-P. Previously reported imaging findings of IOPN-P include large multifocal cysts with MPD dilatation, which are frequently characterized by contrast-enhanced nodules, thickened septa or cyst walls, and growth of a solid component filling more than half of the cyst [6, 7]. Analysis of contrast-enhanced CT and MR characteristics of 18 patients with IOPN-P revealed that MPD dilatation (83.3%) and mural nodule enhancement (62.5%) were frequently observed [6]. Based on T2- and T1-weighted MRI findings, IOPN-P has been reported to be predominantly hyperintense on T2-weighted images and hypointense on T1-weighted images [6, 7]. Although these findings are generally consistent with our cases, there were only a few cases in which the solid component occupied more than half of the cyst (Figure 1).

IOPN-P had a higher frequency and a larger size of solid parts than IPMA. However, IPMA had a higher frequency of dilatation of the downstream MPDs than IOPN-P (Fig. 6). This may reflect the fact that IPMA has a higher mucus-producing capacity and is operated because of the strong MPD dilatation rather than the presence of a solid component. However, some patients with IOPN-P showed marked dilatation of the downstream MPD (Figure 4). This may be due to the potential coexistence of IPMN and IOPN-P pathologies and the presence of components that exhibit high levels of mucus secretion [5, 11]. Radiological peripancreatic invasion and postoperative recurrence were more frequent in IPMC than in IOPN-P (Fig. 7). IPMC infiltrates the pancreatic stroma more often than IOPN-P and is more likely to recur [12]. These findings can be indicative of the clinical and pathological characteristics of IOPN-P, which often exhibits high-grade atypia, but shows limited invasiveness and low metastatic potential [7, 13]. Therefore, if there are imaging findings of peripancreatic invasion, IPMC is more suspected than IOPN. However, IOPN-P invaded the surrounding organs in two patients (Figure 5).

Although previous reports on FDG-PET findings of IOPN-P are limited, IOPN-P shows high FDG accumulation due to activated glucose metabolism in the mitochondria that are abundant in the cytoplasm [14, 15]. However, another study suggested that this was simply due to the large size of the tumor [16]. The SUVmax value of IOPN-P in the present study was 7.5 on average. This finding is consistent with previous reports of FDG-PET of IOPN-P and may reflect active glucose metabolism in cytoplasmically abundant mitochondria. Notably, FDG accumulation is not limited to IOPN-P; IPMC has high FDG accumulation [17, 18]. However, because the prognosis of patients with IOPN-P is better than that of patients with IPMC, even with high FDG accumulation, high FDG accumulation may not indicate high clinical malignancy in patients with IOPN-P.

Serum CEA and CA19-9 levels were significantly lower in IOPN-P than in IPMA and IPMC. However, there were no significant differences among the three groups when normal values were used as the cutoff values. Serum CEA levels have been reported to predict IPMN aggressiveness [19]. However, because CEA levels may increase in the adult population without malignancies [20], it may be difficult to determine clinical significance in differences near low values, as in our cases.

This study has some limitations. First, this was a retrospective study with a small number of patients. Selection bias could not be excluded because only cases of suspected malignancy were resected. Second, there was no uniform imaging protocol for image acquisition owing to the use of multiple scanners from different manufacturers across multiple institutions. In addition, ^18^F-FDG PET was performed in only seven patients. Third, because our study included some IOPN-Ps with partial IPMA pathology, the images may not reflect pure IOPN-P findings. Fourth, only one radiologist blindly evaluated the images; however, a 10-year-experienced radiologist also evaluated all the images and supervised the radiologist. Furthermore, the imaging findings used in this study are those commonly used in clinical practice and no novel imaging findings were utilized; hence, we believe that this has not significantly impacted the results of the evaluation.

## Conclusion

Patients with IOPN-P have a more favorable prognosis than those with IPMC. Imaging findings suggestive of IOPN-Ps include cystic and solid morphology, absence of peripancreatic invasion, large cyst size, and high FDG uptake. In addition, high serum CEA and CA19-9 levels may indicate IPMA or IPMC rather than IOPN-P. However, the preoperative imaging diagnosis of IOPN-P remains challenging, and further studies are required to determine its nature.
